# Circulating PCSK9 as a prognostic biomarker of cardiovascular events in individuals with type 2 diabetes: evidence from a 16.8-year follow-up study

**DOI:** 10.1186/s12933-023-01948-8

**Published:** 2023-08-24

**Authors:** Massimiliano Ruscica, Chiara Macchi, Angelica Giuliani, Alessandra Stefania Rizzuto, Deborah Ramini, Matilde Sbriscia, Stefano Carugo, Anna Rita Bonfigli, Alberto Corsini, Fabiola Olivieri, Jacopo Sabbatinelli

**Affiliations:** 1 Department of Pharmacological and Biomolecular Sciences “Rodolfo Paoletti”, Università degli Studi di Milano, Milan, Italy; 2 Department of Cardio-Thoracic-Vascular Diseases, Foundation IRCCS Cà Granda Ospedale Maggiore Policlinico, Milan, Italy; 3 Department of Clinical and Molecular Sciences, Università Politecnica Delle Marche, Via Tronto 10/A, 60126 Ancona, Italy; 4 Department of Clinical Sciences and Community Health, Università degli Studi di Milano, Milan, Italy; 5 Clinic of Laboratory and Precision Medicine, IRCCS INRCA, Ancona, Italy; 6 Scientific Direction, IRCCS INRCA, Ancona, Italy; 7 Laboratory Medicine Unit, Azienda Ospedaliero Universitaria Delle Marche, Ancona, Italy

**Keywords:** Proprotein Convertase Subtilisin/kexin type 9, Type 2 diabetes mellitus, Cardiovascular risk, Major adverse cardiovascular events, All-cause mortality

## Abstract

**Background:**

Atherosclerotic cardiovascular disease (ASCVD) is the leading cause of morbidity and mortality, being twofold to fourfold more common in patients with type 2 diabetes mellitus (T2DM) than in individuals without diabetes. However, despite this decade-old knowledge, the identification of a specific prognostic risk biomarker remains particularly challenging.

**Methods:**

Taking advantage of a large sample of Caucasian patients (n = 529) with a diagnosis of T2DM followed for a median of 16.8 years, the present study was aimed at testing the hypothesis that fasting serum proprotein convertase subtilisin/kexin type 9 (PCSK9) levels could be prognostic for major adverse cardiovascular events (MACE) and all-cause mortality.

**Results:**

Median levels of PCSK9 were 259.8 ng/mL, being higher in women compared to men and increasing even more in the presence of a complication (*e.g.*, diabetic kidney disease). PCSK9 positively correlated with markers of blood glucose homeostasis (*e.g.*, HbA1c, fasting insulin and HOMA-IR) and the atherogenic lipid profile (*e.g.*, non-HDL-C, apoB and remnant cholesterol). Serum PCSK9 predicted new-onset of MACE, either fatal or non-fatal, only in women (Odds Ratio: 2.26, 95% CI 1.12–4.58) and all-cause mortality only in men (Hazard Ratio: 1.79, 95% CI 1.13–2.82).

**Conclusions:**

Considering that up to two-thirds of individuals with T2DM develop ASCVD in their lifetime, the assessment of circulating PCSK9 levels can be envisioned within the context of a biomarker-based strategy of risk stratification. However, the sex difference found highlights an urgent need to develop sex-specific risk assessment strategies.

*Trial registration*: It is a retrospective study.

**Supplementary Information:**

The online version contains supplementary material available at 10.1186/s12933-023-01948-8.

## Introduction

Type 2 diabetes mellitus (T2DM) has been identified by the United Nation and World Health Organization as one of the five priority non-communicable diseases. Estimates from 2021 indicate that roughly 537 million individuals worldwide have T2DM, a figure that is expected to grow by 46% to 738 million by 2045 [1]. Although progress has been made in promoting population health and extending life expectancy, diabetes reduces global health adjusted life expectancy [2]. Among the most common disabilities related to T2DM, atherosclerotic cardiovascular diseases (ASCVD) is the leading cause of morbidity and mortality, being twofold to fourfold more common in T2DM patients than in individuals without diabetes [3]. Estimates from epidemiological studies report that up to two-thirds of individuals with T2DM develop ASCVD in their lifetime [4], with many events attributable to ischemic heart disease. Hoffner’s study demonstrated that diabetic patients without previous myocardial infarction had as high a risk of myocardial infarction as nondiabetic patients with previous myocardial infarction [5]. Within this context, a population-based autopsy study concluded that the prevalence of coronary atherosclerosis was higher among diabetic individuals than among nondiabetic individuals [6].

Considering that atherosclerosis is the principal cause of ASCVD, the role of proprotein convertase subtilisin/kexin type 9 (PCSK9) cannot be overlooked. The hypothesis that PCSK9 can be directly linked to atherogenesis is supported by observations that PCSK9 is expressed in human atherosclerotic plaques and directly increases atherosclerotic lesion inflammation through a cholesterol-independent mechanism [7, 8]. In line with this evidence, ATHEROREMO-IVUS (The European Collaborative Project on Inflammation and Vascular Wall Remodeling in Atherosclerosis—Intravascular Ultrasound) study showed that the higher the levels of PCSK9, the higher the necrotic core fraction in coronary atherosclerosis. This finding was independent of variation in low-density lipoprotein cholesterol (LDL-C) [9]. PCSK9 is also a regulator of vascular inflammation and its expression correlates with pro-inflammatory cytokine release, inflammatory cell recruitment and plaque destabilization [10]. Preclinical studies showed that PCSK9 overexpression was proatherogenic [11], whereas its absence was protective [12]. Although the pharmacological inhibition of PCSK9 has led to indisputable benefits in terms of cardiovascular (CV)-risk lowering [13], the validity of clinical measurements of circulating PCSK9 for CV-risk prediction, CV- and all-cause mortalities remains an open question [14]. In addition, concerning these last (CV- and all-cause deaths), data from interventional trials (with monoclonal antibodies) [15, 16] or genetic studies (associating  low LDL-C to *PCSK9* variants) [17] are contrasting.

Despite the decade-old knowledge that T2DM is a major risk factor for ASCVD, the reasons for this association are only partially understood [18]. The risk of ASCVD in T2DM depends largely on the concomitant presence of traditional CV risk factors including LDL-C, high blood pressure and smoking, although they do not fully explain the heterogeneity [19]. This aspect poses a particular challenge in the identification of a prognostic risk biomarker. In the case of T2DM, the evaluation of PCSK9 as a prognostic tool of CV events seems to be inconsistent and to depend on patient-level CV risk and background treatment [20]. Thus, taking advantage of a large sample of Caucasian patients (n = 529) with T2DM followed for a median of 16.8 years, the present study was aimed at testing the hypothesis that fasting PCSK9 levels could be prognostic for major adverse cardiovascular events (MACE) and all-cause mortality.

## Methods

### Study subjects

Samples were collected from an extensively characterized cohort of 568 patients diagnosed with T2DM [21]. The patients were recruited at the Metabolic Diseases and Diabetology Department of IRCCS INRCA between May 2003 and November 2006. For the current investigation, 529 T2DM patients (median age = 67 years, interquartile range 62–72 years) were included. T2DM was diagnosed based on the criteria established by the American Diabetes Association, which include a hemoglobin A1C (HbA1C) level of ≥ 6.5%, fasting blood glucose level of ≥ 126 mg/dl, 2-h blood glucose levels of ≥ 200 mg/dl after an oral glucose tolerance test (OGTT), or a random blood glucose level of ≥ 200 mg/dl in the presence of severe diabetes symptoms [22]. Patients with diabetes were eligible for inclusion if they had a body mass index (BMI) of ≤ 40 kg/m^2^, were between the ages of 40 and 87 years, and were able and willing to provide written informed consent. Fasting blood samples from all participants were processed for serum separation and stored at a temperature of −80 °C.

### Ethics statement

The study received approval from the Institutional Review Board of IRCCS INRCA hospital (Approval No. 34/CdB/03), and written informed consent was obtained from each participant in accordance with the principles outlined in the Declaration of Helsinki.

### Assessment of serum PCSK9

Serum concentrations of PCSK9 were measured by a commercial ELISA kit (R&D Systems, MN, USA). Samples were diluted at 1:20 and incubated onto a microplate pre-coated with a monoclonal human-PCSK9-specific antibody. Sample concentrations were obtained by a four-parameter logistic curve-fit, with a minimum detectable PCSK9 concentration of 0.219 ng/mL [23]. Intra- and inter-assay coefficients of variability were 4.9 ± 1.4% and 5.4 ± 1.5%, respectively.

### Outcomes

The study assessed outcome events, specifically the occurrence of new MACE in patients who did not have a history of MACE at the time of enrolment, as well as all-cause mortality. MACE was defined as the occurrence of nonfatal events, namely, myocardial infarction, cardiac arrest, cardiogenic shock, life-threatening arrhythmia, or stroke. Information on outcomes was collected by reviewing medical records, starting from the date of enrolment (May 2003 to November 2006) and continuing until the last day of follow-up (December 31st, 2019).

### Covariates

Data regarding vital signs, anthropometric measurements, medical history, behaviours, exercise habits, and concurrent treatments were collected for all participants. Standard procedures were employed to evaluate blood cell count and biochemical variables in all subjects. The estimated glomerular filtration rate (eGFR) was determined using the CKD-EPI (Chronic Kidney Disease Epidemiology Collaboration) equation, taking into account serum creatinine, age, sex, and ethnicity. The presence of diabetic complications was determined as previously described [24]. Diabetic retinopathy was assessed by fundoscopy through dilated pupils and/or fluorescence angiography. Incipient nephropathy was characterized by a urinary albumin excretion rate greater than 30 mg/g creatinine and a normal creatinine clearance. Neuropathy was diagnosed using electromyography. Ischemic heart disease was determined based on clinical history and/or ischemic electrocardiographic abnormalities. Peripheral artery disease, including atherosclerosis obliterans and cerebrovascular disease, was identified through physical examinations and Doppler velocimetry.

### Statistical analysis

Continuous variables were presented as mean and standard deviation or median and interquartile range, depending on their distribution, which was assessed using the Shapiro–Wilk test. To compare serum PCSK9 levels among groups, the Mann–Whitney U test and Kruskal–Wallis test followed by Dunn post-hoc test were utilized. Categorical variables were compared using the χ^2^ test. Spearman's correlation was employed to examine the associations between continuous variables. Two-way analysis of variance (ANOVA) was conducted to explore sex-related differences in the serum levels of PCSK9 among groups. Multivariable analysis of covariance (ANCOVA), with Tukey's *post hoc* tests, was performed using PCSK9 concentrations as the dependent variable, T2DM complications as factors, and age, sex, and HbA1c as covariates to identify factors associated with T2DM complications and treatments. The association between PCSK9 levels and follow-up endpoints was investigated using Kaplan–Meier curves and Cox proportional hazards analysis, adjusted for sex, age, hypertension, therapy with statins or with vitamin K antagonists, smoking status, disease (T2DM) duration, HbA1c, BMI, high–sensitivity C-reactive protein (hs-CRP), non-high-density lipoprotein cholesterol (non-HDL-C), and eGFR, with 95% confidence intervals.

Logistic regressions were used to assess associations with MACE, as the precise timing of most events could not be determined. Optimal sex-specific PCSK9 cut-offs for predicting survival in T2DM patients were determined using the Evaluate Cutpoints R package [25]. The package uses maximally selected rank statistics and Cox proportional hazard model to compute the best cut-off for a given continuous predictor of a survival function. Biomarkers were included in the models as either continuous or categorized variables. No missing data were present for the covariates of interest. The investigators had full access to the database population used to create the study population.

Statistical significance was defined as p < 0.05. Data analysis was performed using R (version 4.1), the Jamovi software (version 2.3.1), and SPSS 28.0 for Windows software (SPSS Inc.; Chicago, IL, USA).

## Results

Serum samples from a total of 529 patients with T2DM were analysed. Baseline subject’s characteristics are reported in Table 1. After a median follow-up of 16.8 years (interquartile range, 13.0–16.8), 196 patients died (37.1%). The mean survival time was 14.2 (95% CI 13.8–14.6) years. At the time of enrolment, 289 (54.6%) patients had had at least one complication. Among 240 patients (45.4%) with uncomplicated T2DM at the time of enrolment, 149 patients (67.4%) developed at least one complication. Survival was higher in T2DM patients without complications compared to patients with at least one complication (log-rank test, p < 0.0001; Additional file 1: Figure S1).

**Table 1:** Biochemical and anthropometric characteristics of patients with type 2 diabetes mellitus

Variables	T2DM N = 529
Age (years)	67 (62–72)
Sex (Males, %)	280 (53%)
Current smoking (n, %)	78 (15%)
BMI (Kg/m^2^)	28.1 (25.8–31.4)
Weight (Kg)	77 (69–86)
Waist-hip ratio	0.94 (0.89–0.98)
Total cholesterol (mg/dL)	204 (181–233)
LDL-C (mg/dL)	114 (96–136)
HDL-C (mg/dL)	50 (42–60)
non-HDL-C (mg/dl)	152 (130–177)
Triglycerides (mg/dL)	115 (83–159)
Remnant cholesterol (mg/dL)	34 (23–50)
ApoB (mg/dL)	100 (84–118)
ApoA1 (mg/dL)	164 (146–186)
PCSK9 (ng/mL)	260 (211–305)
Fasting glucose (mg/dL)	153 (133–183)
HbA1C (%)	7.3 (6.6–8.0)
Insulin (µUI/mL)	5.69 (3.66–8.62)
HOMA index	2.14 (1.39–3.56)
Hemoglobin (g/dL)	14.2 (13.4–15.2)
WBC (n/mm^3^)	6.57 (5.50–7.54)
Platelets (n/mm^3^)	209 (179–252)
hs-CRP (mg/L)	2.51 (1.23–4.65)
Fibrinogen (mg/dL)	298 (255–344)
Iron (µg/dL)	81 (64–96)
Ferritin (ng/mL)	88 (46–160)
Creatinine (mg/dL)	0.90 (0.70–1.00)
eGFR (mL/min)	81 (66–86)
Azotemia (mg/dL)	38 (32–46)
Uric acid (mg/dL)	4.6 (4.1–5.4)
Alanine aminotransferase (U/L)	39 (33–47)
Aspartate aminotransferase (U/L)	20 (16–24)
Total bilirubin (mg/dL)	0.6 (0.5–0.8)
Gamma-glutamyl transferase (U/L)	49 (39–61)
Disease duration (years)	14 (7–23)
*Relevant medications (n, %)*	
Any T2DM medication	403 (76%)
Metformin	196 (37%)
Sulphonylureas	257 (49%)
Glinides	12 (2%)
Insulin	95 (18%)
Statins	103 (19%)
Vitamin K antagonists	52 (10%)
*T2DM complications (n, %)*	
Retinopathy	149 (28%)
Nephropathy	68 (13%)
Neuropathy	98 (19%)
History of MACE	79 (15%)
Peripheral artery disease	50 (9%)

Data are presented as median (interquartile range) or number (%) for continuous and categorical variables, respectively

*Apo* apolipoprotein, *BMI* body mass index, *eGFR* estimated glomerular filtration rate, *HbA1C* hemoglobin A1C, *HDL-C* high-density lipoprotein cholesterol, *HOMA* Homeostasis model assessment for Insulin Resistance, *hs-CRP* high-sensitivity C-reactive protein, *MACE* major adverse cardiovascular events, *LDL-C* low-density lipoprotein cholesterol, *PCSK9* proprotein convertase subtilisin/kexin type 9, *WBC* white blood cells, *T2DM* type 2 diabetes mellitus

**Fig. 1:**
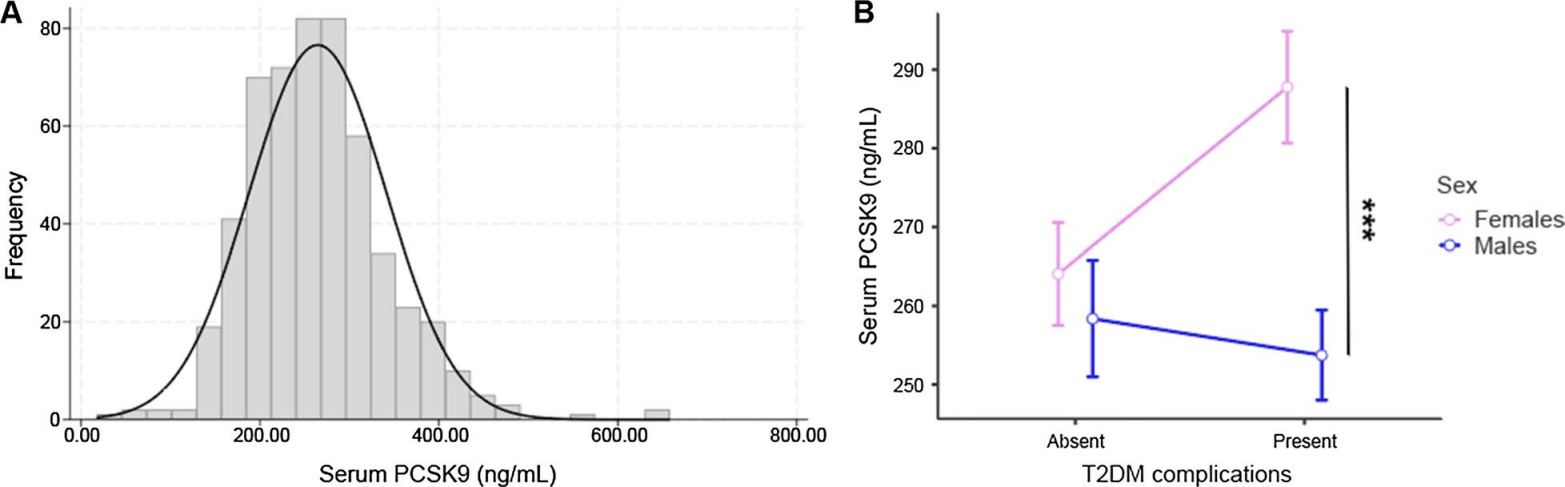
*Assessment of circulating levels of PCSK9*. **A** Distribution plot of serum PCSK9 concentrations in patients with T2DM. **B** Marginal means plot of serum PCSK9 in patients with T2DM grouped according to sex and presence of complications. ***p < 0.001 for Tukey’s post-hoc tests following two-way ANOVA. *PCSK9* proprotein convertase subtilisin/kexin type 9, *T2DM* type 2 diabetes mellitus

Figure 1A shows the distribution of serum PCSK9 in our cohort, which is moderately right-skewed (coefficient of skewness = 0.77). Median levels were 259.8 ng/mL and ranged from 17.7 to 657.9 ng/mL (interquartile range, 210.5–305.2 ng/mL). Serum PCSK9 was higher in females (median: 271.3 ng/mL [interquartile range: 221.9–317.8 ng/mL] vs. 246.9 ng/mL [interquartile range: 204.2– 298.8 ng/mL], p = 0.001), and this difference was particularly evident in those carrying at least one T2DM complication. The impact of sex on the levels of PCSK9 was even more evident when at least one complication was present, namely, higher levels PCSK9 were found in women with at least one complication compared to women without additional complications and compared to males with either uncomplicated (p = 0.022) or complicated (p < 0.001) T2DM (Fig. 1B, Additional file 1: Table S1). On this matter, a significant interaction between sex and presence of complications stands in determining PCSK9 levels (p = 0.035). Conversely, sex-related differences are not present in patients with uncomplicated diabetes (p = 0.939). Age did not seem to impact PCSK9 levels, in both sexes (males, Spearman’s rho = 0.059, p = 0.322; females, Spearman’s rho = 0.066, p = 0.304).

**Fig. 2:**
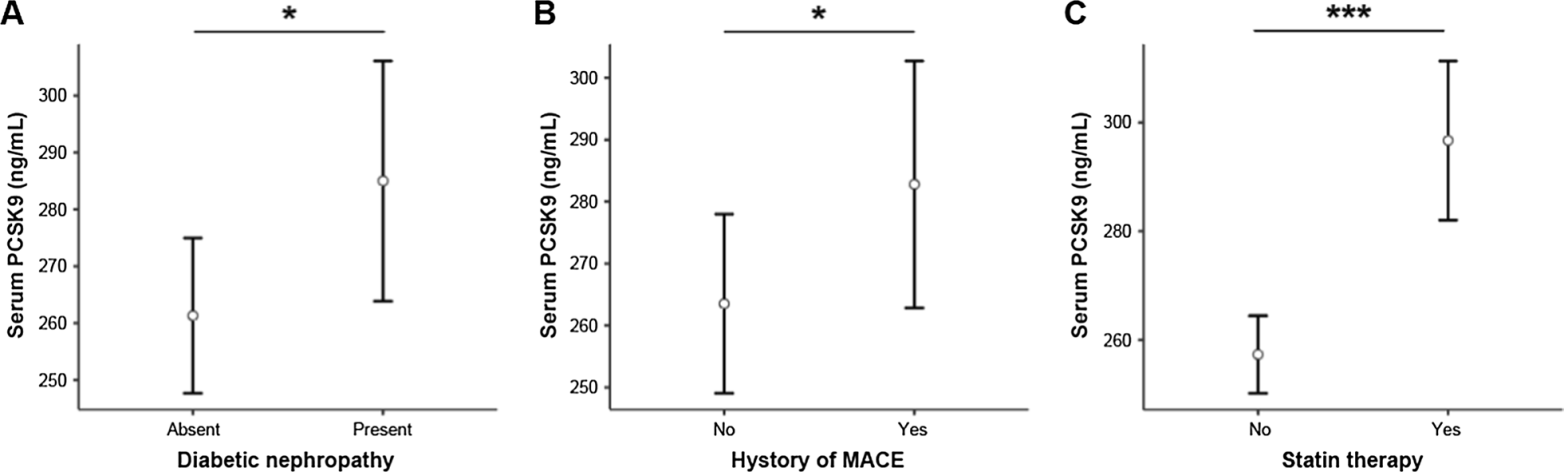
*Levels of PCSK9 according to complications*. Marginal means plot of serum PCSK9 in patients with type 2 diabetes mellitus  grouped according to presence of **A** diabetic nephropathy, **B** history of MACE and **C** statin therapy, sex and presence of complications. *p < 0.05; ***p < 0.001 for Tukey’s *post-hoc* tests following one-way ANCOVA. *MACE* major adverse cardiovascular events, *PCSK9* proprotein convertase subtilisin/kexin type 9

To further explore whether complications associated to T2DM affected the circulating levels of PCSK9, an ANCOVA test, followed by Tukey’s *post-hoc* comparisons, was computed after adjustment for age, sex, HbA1c, and the presence of previous MACE, atherosclerotic vascular disease, nephropathy, neuropathy and retinopathy. Serum PCSK9 was significantly higher in subjects with diabetic kidney disease (p = 0.020; Fig. 2A) and in patients with a history of MACE (p = 0.043; Fig. 2B), whereas atherosclerotic vascular disease, nephropathy, and neuropathy did not affect PCSK9 levels (Additional file 1: Table S2). No significant association was observed with antidiabetic drugs (*i.e.*, metformin, insulin, sulphonylureas, glinides; Additional file 1: Table S3), whereas PCSK9 levels rose in patients   on  statins, independent of age, sex, LDL-C, and HbA1c (p < 0.001; Fig. 2C; Additional file 1: Table S4).

**Fig. 3:**
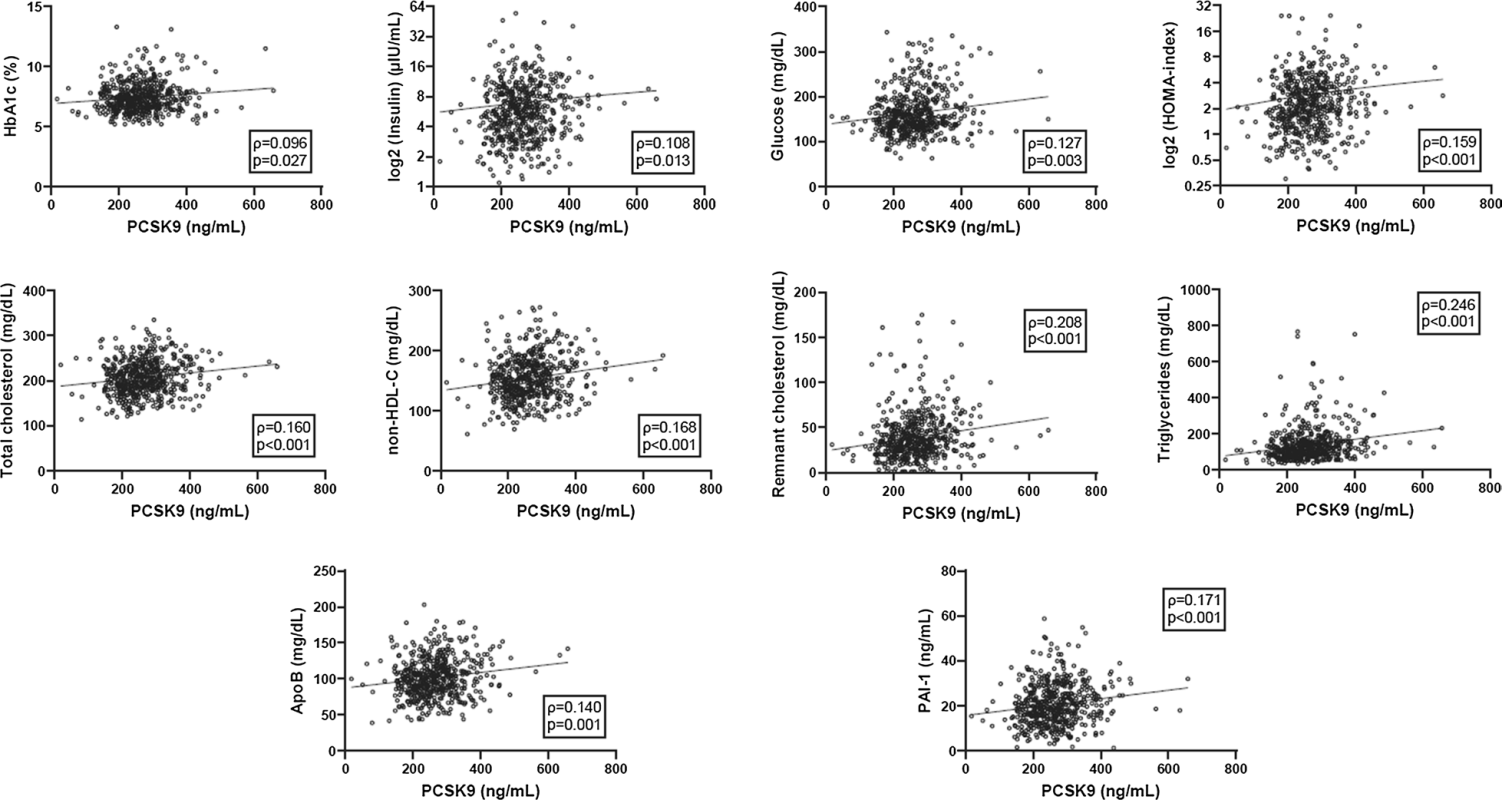
Scatter diagrams. Dot plots showing significant correlations between serum PCSK9 and biochemical covariates. Regression lines are reported. For each correlation, Spearman’s rho coefficients and p-values are reported. *ApoB *apolipoproteinB, *HbA1C* hemoglobin A1C, *HOMA* Homeostasis model assessment for Insulin Resistance, *non-HDL-C* non-high-density lipoprotein cholesterol, *PAI-1* plasminogen activator inhibitor 1, *PCSK9* proprotein convertase subtilisin/kexin type 9

We then explored the correlations between serum PCSK9 and the available biochemical variables. Among the significant Spearman’s correlations, reported in the complete correlation matrix (Additional file 1: Table S5), according to the topic of the present study, it is worth highlighting the positive correlations between PCSK9 and markers of blood glucose homeostasis [HbA1c (p = 0.027), fasting insulin (p = 0.012) and glucose (p = 0.003), HOMA-index (p < 0.001)] and those of lipid profile [total cholesterol (p < 0.001), non-HDL-C (p < 0.001), remnant cholesterol (p < 0.001), triglycerides (p < 0.001), and ApoB (p = 0.001)]. A positive correlation was found also with PAI-1 (p < 0.001). Scatter diagrams of the highlighted correlations are reported in Fig. 3.

### Prognostic value of PCSK9 in T2DM

The next step consisted in evaluating whether serum PCSK9 was able to predict long-term survival (16.8 years) in T2DM patients using Kaplan-Maier and univariable and multivariable Cox regression methods. To achieve this aim, two sex-specific cut-offs for PCSK9 were computed to maximize the differences in survival prediction. The cut-offs for T2DM patients were 244 ng/mL and 299 ng/mL, respectively, for males and females. 51.8% of males and 31.3% of females were above the threshold. PCSK9 levels above the cut-off were associated, at the univariate level, with all-cause mortality only in males, whereas the correlation was lost after adjusting for age, hypertension, therapy with statins or with vitamin K antagonists, smoking status, disease (T2DM) duration, HbA1c, BMI, hs-CRP, non-HDL-C, and eGFR.

**Fig. 4:**
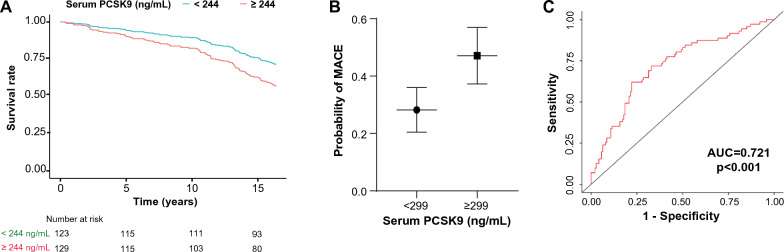
Kaplan-Meir curves. **A** Kaplan–Meier survival estimates for male patients with type 2 diabetes mellitus grouped according to PCSK9 levels. **B** Marginal means plot showing the probability of developing MACE in females with T2DM grouped according to PCSK9 levels. **C** ROC curve for the logistic regression model. *AUC* area under the curve, *MACE* major adverse cardiovascular events, *PCSK9 *proprotein convertase subtilisin/kexin type 9, *ROC* receiving operating characteristic curve

The association was statistically significant also at the multivariable analysis (Hazard Ratio (HR): 1.79, 95% CI 1.13–2.82) when considering only subjects aged  ≤ 75 years (Table 2). The corresponding Kaplan–Meier curves are displayed in Fig. 4A. In women, serum PCSK9 did not associate with all-cause mortality (Table 2), also in patients  ≤ 75 years (data not shown).

**Table 2:** Cox regression analysis of serum PCSK9 for the prediction of all-cause mortality in patients with type 2 diabetes mellitus

Predictor	Males			Males ≤ 75 yrs			Females		
	N (%)	HR (univariable)	HR (multivariable)	N (%)	HR (univariable)	HR (multivariable)	N (%)	HR (univariable)	HR (multivariable)
PCSK9 ≥ 244 ng/mL	144 (52.0)	**1.60 (1.09–2.35)**	1.25 (0.90–2.03)	129 (51.6)	**1.99 (1.30–3.06)**	**1.79 (1.13–2.82)**	–	–	–
PCSK9 ≥ 299 ng/mL	–	–	–	–	–	–	78 (31.3)	1.33 (0.85–2.08)	1.08 (0.67–1.72)
Current smoking	41 (14.8)	–	1.71 (1.00–2.92)	39 (15.6)	–	**1.87 (1.07–3.27)**	37 (14.9)	–	1.26 (0.63–2.54)
Hypertension	179 (64.6)	–	1.27 (0.81–1.98)	159 (63.6)	–	1.24 (0.77–2.02)	166 (66.9)	–	1.11 (0.67–1.83)
Statin therapy	51 (18.4)	–	0.64 (0.38–1.10)	47 (18.8)	–	0.67 (0.38–1.18)	52 (21.0)	–	1.31 (0.67–2.18)
Vitamin K antagonist therapy	28 (10.1)	–	1.04 (0.56–1.90)	26 (10.4)	–	0.94 (0.48–1.83)	24 (9.7)	–	1.24 (0.62–2.47)
Disease duration (years)	277	–	1.01 (0.99–1.03)	250	–	1.01 (1.00–1.03)	248	–	1.01 (0.99–1.04)
Age (years)	277	–	**1.10(1.06**–**1.14)**	250	–	**1.10 (1.05**–**1.14)**	248	–	**1.11 (1.07**–**1.16)**
HbA1c (%)	277	–	1.16 (1.00–1.34)	250	–	1.12 (0.95–1.32)	248	–	1.18 (0.95–1.46)
hs-CRP (mg/L)	277	–	1.01 (0.98–1.04)	250	–	1.00 (0.97–1.03)	248	–	1.03 (1.00–1.05)
non-HDL-C (mg/dL)	277	–	1.00 (0.99–1.00)	250	–	1.00 (0.99–1.01)	248	–	1.00 (0.99–1.01)
BMI (kg/m^2^)	277	–	1.05 (1.00–1.12)	250	–	1.06 (1.00–1.13)	248	–	0.98 (0.94–1.03)
eGFR (mL/min)	277	–	0.99 (0.98–1.00)	250	–	0.99 (0.98–1.00)	248	–	0.99 (0.98–1.00)

*BMI* body mass index, *eGFR* estimated glomerular filtration rate, *HbA1c* hemoglobin A1C, *HDL-C* high-density lipoprotein cholesterol, *hs-CRP* high-sensitivity C-reactive protein, *PCSK9* proprotein convertase subtilisin/kexin type 9

N = numerosity. Crude and adjusted hazard ratios (HR) with 95% confidence intervals are shown

Significant predictors are in bold

Then, the prognostic value of PCSK9 was tested for MACE, either fatal or non-fatal, in individuals with T2DM but no prior history of MACE. In this subgroup of 450 subjects, 151 MACE (36%) were recorded during the follow-up period, 71 in females and 80 in males. A binomial logistic regression adjusted for the most relevant clinical and biochemical predictors showed that serum PCSK9 ≥ 299 ng/mL was associated with increased odds of MACE in females (Odds Ratio (OR): 2.26, 95% CI 1.12–4.58) (Table 3). Conversely, no association between PCSK9, either continuous or categorized, and MACE was found in males. The probability of developing the outcome according to the score is shown in Fig. 4B, whereas Fig. 4C depicts the ROC curve for the model (accuracy, 70.1%; sensitivity, 33.8%; specificity, 88.6%; area under the curve (AUC): 0.721).

**Table 3:** Logistic regression model predicting likelihood of developing major adverse cardiovascular events (MACE) in patients with type 2 diabetes mellitus without previous history of MACE

Predictor	Males			Females		
	N (%)	Estimate (SE)	OR (95% CI)	N (%)	Estimate (SE)	OR (95% CI)
PCSK9 ≥ 244 ng/mL	105 (47)	−0.376 (0.327)	0.69 (0.36–1.30)	–	–	–
PCSK9 ≥ 299 ng/mL	–	–	–	65 (29)	0.816 (0.360)	**2.26 (1.12**–**4.58)**
Current smoking	36 (16)	−0.122 (0.449)	0.89 (0.37–2.13)	34 (15)	0.425 (0.448)	1.53 (0.64–3.68)
Hypertension	135 (60)	0.147 (0.340)	1.16 (0.60–2.25)	145 (65)	0.947 (0.369)	2.58 (1.25–5.31)
Statin therapy	29 (13)	0.477 (0.461)	1.61 (0.65–3.98)	45 (20)	−0.070 (0.403)	0.93 (0.42–2.05)
Vitamin K antagonist therapy	19 (8)	2.197 (0.682)	**8.99 (2.36**–**34–2)**	21 (9)	0.239 (0.539)	1.27 (0.44–3.65)
Disease duration (years)	226	0.007 (0.015)	1.01 (0.98–1.04)	224	−0.020 (0.018)	0.98 (0.95–1.01)
Age (years)	226	0.022 (0.022)	1.02 (0.98–1.07)	224	0.055 (0.026)	**1.06 (1.01**–**1.11)**
HbA1c (%)	226	0.125 (0.131)	1.13 (0.88–1.47)	224	0.229 (0.153)	1.26 (0.93–1.70)
hs-CRP (mg/L)	226	0.008 (0.025)	1.01 (0.96–1.06)	224	0.001 (0.035)	1.00 (0.93–1.07)
non-HDL-C (mg/dL)	226	0.006 (0.005)	1.01 (0.99–1.02)	224	−0.003 (0.005)	1.00 (0.99–1.01)
BMI (kg/m^2^)	226	−0.029 (0.043)	0.97 (0.89–1.06)	224	−0.010 (0.036)	0.99 (0.92–1.06)
eGFR (mL/min)	226	0.013 (0.009)	1.01 (0.99–1.03)	224	−0.013 (0.009)	0.99 (0.97–1.00)

Odds Ratio (OR) with 95% confidence interval (CI) are shown. *SE* standard error

*BMI* body mass index, *eGFR* estimated glomerular filtration rate, *HbA1c* hemoglobin A1C, *HDL-C* high-density lipoprotein cholesterol, *hs-CRP* high-sensitivity C-reactive protein, *PCSK9* proprotein convertase subtilisin/kexin type 9

Model summary, males, χ^2^ = 23.1, df = 12, p < 0.027, Nagelkerke’s R^2^ = 0.141; females, χ^2^ = 28.3, df = 12, p = 0.005, Nagelkerke’s R^2^ = 0.174

Significant data are in bold

## Discussion

The main finding of the present study relates to the ability of PCSK9 to predict, during a long-term follow-up (16.8 years), new-onset of MACE (both fatal and non-fatal) and all-cause mortality in a sex-specific manner. PCSK9 associated with the occurrence of myocardial infarction, cardiac arrest, cardiogenic shock, life-threatening arrhythmia, or stroke only in women, whereas it associated with death only in men.

A substantial proportion of patients with T2DM with established ASCVD or multiple risk factors have evidence of ongoing myocardial injury, hemodynamic stress, or systemic inflammation [26]. Thus, it becomes important to identify risk stratification biomarkers that can mirror the underlying aetiologies of atheroma formation. Although loss-of-function variations in *PCSK9* confer protection against cardiovascular heart disease [27–29] and pharmacological inhibition of PCSK9 appears to be effective in reducing the risk of MACE also in subjects with diabetes [30], the value of PCSK9 level measurements as a prognostic biomarker for MACE prediction remains a field fraught with uncertainty. The results of a Swedish prospective study enrolling 4232 men and women aged 60 years were prominent in this area. Baseline serum PCSK9 concentration predicted incidence of ASCVD events during a follow-up of 15 years [31]. In line with this evidence, ATHEROREMO-IVUS study showed that the higher the PCSK9 levels, the higher the necrotic core fraction in coronary atherosclerosis. This finding was independent of variation in LDL-C [9]. PCSK9 levels were also accurate when used to predict acute coronary syndrome (ACS) at 24-month follow-up in patients with severe carotid artery atherosclerosis undergoing carotid endarterectomy. Specifically, PCSK9 values > 431.3 ng/mL were correlated with a higher risk of occurrence of ACS [32]. In patients with coronary artery disease undergoing percutaneous coronary intervention (PCI), during a follow-up of 28.4 months, baseline PCSK9 levels were associated with MACE and mortality [33]. In line with this evidence, among patients with ST-segment elevation myocardial infarction undergoing PCI, those with high PCSK9 levels and diabetes mellitus had the lowest cumulative event-free survival rate [34]. Conversely, among 358 women who subsequently developed MACE, during a 17-year follow-up, baseline levels of PCSK9 did not predict the first cardiovascular event [35]. Similar conclusions were reached by Gencer et al. in ACS patients undergoing coronary angiography. PCSK9 was clearly associated with inflammation and hypercholesterolemia, but did not predict mortality at one year [36]. Likewise, the ability of PCSK9 to predict CV events was not demonstrated in kidney transplant candidates [37].

Relative to T2DM, data are inconsistent. A *post hoc* analysis of the DIABHYCAR (Non-insulin Dependent Diabetes, Hypertension, Microalbuminuria or Proteinuria, Cardiovascular Events and Ramipril) cohort and SURDIAGENE (Survie, Diabète de type 2 et Genétique) cohort showed that the predictivity of CV events captured by PCSK9 depended on the individuals’ CV risk. The association was lost in T2DM patients with a high CV risk [20]. Differently from these two studies, with a follow-up of 4.5 years and 6.6 years, respectively, our results are based on a very long-term follow-up (a median of 16.8 years), that, to the best of our knowledge, is the longest so far described. Our results showed not only that PCSK9 increases according to the complications associated with T2DM (*e.g.*, previous MACE), but also that PCSK9 has the ability to predict the early onset of MACE, although in a sex-specific manner (only in women). In the vast majority of ASCVD, there are differences between women and men in epidemiology, pathophysiology, clinical manifestations, effects of therapy and outcomes [38]. On this matter, many studies described PCSK9 plasma levels to be significantly higher in females than in males [39, 40]. It is worth mentioning that T2DM is a stronger risk factor for certain ASCVD events in women than in men as reported in the INTERHEART study [41]. Similar results arose from the analysis of the 20-year follow-up of the Framingham Heart Study showing that T2DM was associated with a similar risk of all ASCVD events in women and men, with, however, a greater risk of coronary heart disease and ASCVD death in women [42]. The existence of a close relationship between lipid and glucose metabolism has prompted us to check a possible correlation between PCSK9 and glucose homeostasis. We found that PCSK9 levels were significantly and positively correlated with insulin, glucose, HOMA-IR and HbA1c. Although the correlation between PCSK9 and glucose homeostasis is consistent across a large range of studies [43, 44], it is worth highlighting that data from seven prospective, randomized trials involving serial coronary intravascular ultrasonography concluded that HbA1c levels were independently associated with MACE [45]. In recent years a gradient of mortality risk with increasing HbA1c > 6–6.9% has been observed, suggesting that HbA1c remains an informative predictor of outcomes even if causality cannot be inferred [46].

 Concerning all-cause mortality, data on PCSK9 are scanty and not concordant. In genetic analyses, *PCSK9* variants (associated to low LDL-C) were not causally associated with low all-cause mortality (at least in the general population) [17], whereas a Bayesian network meta-analysis of 54,311 patients in secondary prevention concluded that only alirocumab reduced all-cause mortality [47]. Overall, discrepancies might be explained by differences in the frequency of ASCVD in the studied populations. Reducing CV mortality in a population with a low frequency of ASCVD will have little effect on all-cause mortality, conversely a similar reduction in risk of CV mortality in a population with a high frequency of ASCVD disease is more likely to translate into a risk reduction in all-cause mortality [17]. In patients with heart failure, PCSK9 levels predict the risk of mortality [48]. Besides that, the predictivity of circulating PCSK9 on all-cause mortality were in line with a previous 3-year follow-up study on haemodialysis patients showing that PCSK9 was independently associated with all-cause mortality [49]. Although the association remained valid also when people < 75 years were considered, we have no explanation for this sex-specific effect.

Concerning lipid metabolism, the positive correlations between PCSK9 and non-HDL-C, apoB and remnant cholesterol become of interest considering that our cohort comprises only diabetic individuals. While LDL-C, non-HDL-C, and apoB concentrations are highly correlated, there are clinical scenarios (*e.g.*, in patients with diabetes) where LDL-C might underestimate the concentration of atherogenic apoB-containing lipoproteins. In these individuals, non-HDL-C is a stronger predictor of mortality from coronary disease than LDL-C. Overall, non-HDL-C concentrations in blood are strongly associated with long-term risk of ASCVD [50]. In a *post hoc* analysis of patients with diabetes from four prospective cohort studies, the relative risk of death for diabetic (compared to non-diabetic) patients was 7.2 for those with elevated non-HDL-C and 5.7 for those with low non-HDL-C [51]. Results of a prospective cohort analysis including individuals from the population-based UK Biobank and from 2 large international clinical trials, FOURIER (Further Cardiovascular Outcomes Research with PCSK9 Inhibition in Subjects with Elevated Risk) and IMPROVE-IT (IMProved Reduction of Outcomes: Vytorin Efficacy International Trial), concluded that risk of myocardial infarction was best captured by the number of apoB-containing lipoproteins [52]. Furthermore, apoB resulted to be a more accurate marker of CV risk in statin-treated patients than LDL-C or non-HDL-C [53]. Relative to remnant cholesterol, among 1,956,452 patients with T2DM and without ASCVD, remnant cholesterol was associated with ASCVD, independent of the levels of LDL-C or other conventional ASCVD risk factors [54].

Finally, increased concentrations of PAI-1 in blood are associated with a predisposition toward venous thrombosis and compelling evidence shows markedly increased concentrations of PAI-1 in blood and the arterial wall of individuals with T2DM [55]. Within this context, our positive correlation between PCSK9 and PAI-1 is not surprising considering that genetic deficiency of PAI-1 is associated with reduced plasma PCSK9 levels in humans [56].

These results should be interpreted whilst keeping in mind potential limitations. First, this is a monocentric retrospective study with a single determination of PCSK9 serum levels and, thus, possible fluctuations and different exposures to lipid-lowering drugs could not be considered. Second, none of the patients were treated with the novel antidiabetic drugs (*e.g.*, gliflozin) at the time of enrolment. However, the enrolled T2DM patients were constantly followed by a dedicated facility, with strong adherence to the latest standards of care and with periodic monitoring for the development of complications, as confirmed by the very small proportion of loss to follow-up patients (1.2%). Thirdly, although no intermediate information was available on the degree of blood glucose control and on biochemical variables related to T2DM complications, a potential explanation for the different results compared to previous studies is the lack of a “gold standard” to measure PCSK9. However, the assay we used is highly validated and the intra-assay and inter-assay coefficients of variability ensure good reproducibility as demonstrated by running this assay in roughly 7000 samples in the last years [23, 40, 57–59]. Samples were collected and stored at −80 °C for the entire follow-up period and dedicated serum aliquots were used only for the present study that required 20 μL. The reliability of our results is demonstrated by the well-known effect of sex [40], comorbidities (*e.g.* renal function) [57] and statin treatment [60] on PCSK9 levels. Indeed, in our population PCSK9 levels were raised in women, in those with nephropathy and under statin treatment. A fifth limitation is the absence of a genetic analysis allowing to identify patients with familial hypercholesterolemia. Plasma PCSK9 levels are positively associated with LDL-C levels in carriers of *LDLR* or *APOB* mutations and might contribute to the phenotypic severity of this disorder. [61] Specifically related to PCSK9 mutations, since loss-of-function and particularly gain-of-function mutations are relatively rare in the Caucasian population [27], the exclusion of these subjects from the analysis is predicted to have a minimal impact on the statistical results. Sixthly, we do not have data on the reproductive hormones, although being the median age 67 years, we assume all female patients gained menopause.

In conclusion, considering that up to two-thirds of individuals with T2DM develop ASCVD in their lifetime, the results of this retrospective study highlight the utility of measuring PCSK9 in the context of a biomarker-based strategy of risk stratification. However, the sex difference we noticed highlights an urgent need to develop sex-specific risk assessment strategies.

### Supplementary Information

**Additional file 1: Figure S1.** Kaplan–Meier survival estimates with 95% confidence intervals for patients with type 2 diabetes mellitus grouped according to the absence or presence of complications associated to diabetes. **Table S1.** Two-way ANOVA assessing the effect of sex and the presence of complications on circulating levels of PCSK9. **Table S2.** Circulating levels of PCSK9 in patients with type 2 diabetes mellitus (T2DM) in relation to the different T2DM-related complications. **Table S3.** Circulating levels of PCSK9 in patients with type 2 diabetes mellitus in relation to antidiabetic treatments. **Table S4.** Circulating levels of PCSK9 in patients with type 2 diabetes mellitus in relation to statin therapy. **Table S5.** Correlation matrix between selected biochemical variables and serum PCSK9 in patients with type 2 diabetes mellitus.

## Data Availability

Data are available from the corresponding author on reasonable request.
