# Supplementary material for: Circulating PCSK9 as a prognostic biomarker of cardiovascular events in individuals with type 2 diabetes: evidence from a 16.8-year follow-up study
Source: Cardiovasc Diabetol. 2023 Aug 24;22:222. doi: 10.1186/s12933-023-01948-8 (PMC10464486; doi:10.1186/s12933-023-01948-8)
Supplement: Supplementary file 1 — Additional file 1: Figure S1. Kaplan–Meier survival estimates with 95% confidence intervals for patients with type 2 diabetes mellitus grouped according to the absence or presence of complications associated to diabetes. Table S1. Two-way ANOVA assessing the effect of sex and the presence of complications on circulating levels of PCSK9. Table S2. Circulating levels of PCSK9 in patients with type 2 diabetes mellitus (T2DM) in relation to the different T2DM-related complications. Table S3. Circulating levels of PCSK9 in patients with type 2 diabetes mellitus in relation to antidiabetic treatments. Table S4. Circulating levels of PCSK9 in patients with type 2 diabetes mellitus in relation to statin therapy. Table S5. Correlation matrix between selected biochemical variables and serum PCSK9 in patients with type 2 diabetes mellitus. [file 12933_2023_1948_MOESM1_ESM.docx]

**SUPPLEMENTARY MATERIAL**

**Figure S1.** Kaplan-Meier survival estimates with 95% confidence intervals for patients with type 2 diabetes mellitus grouped according to the absence or presence of complications associated to diabetes.


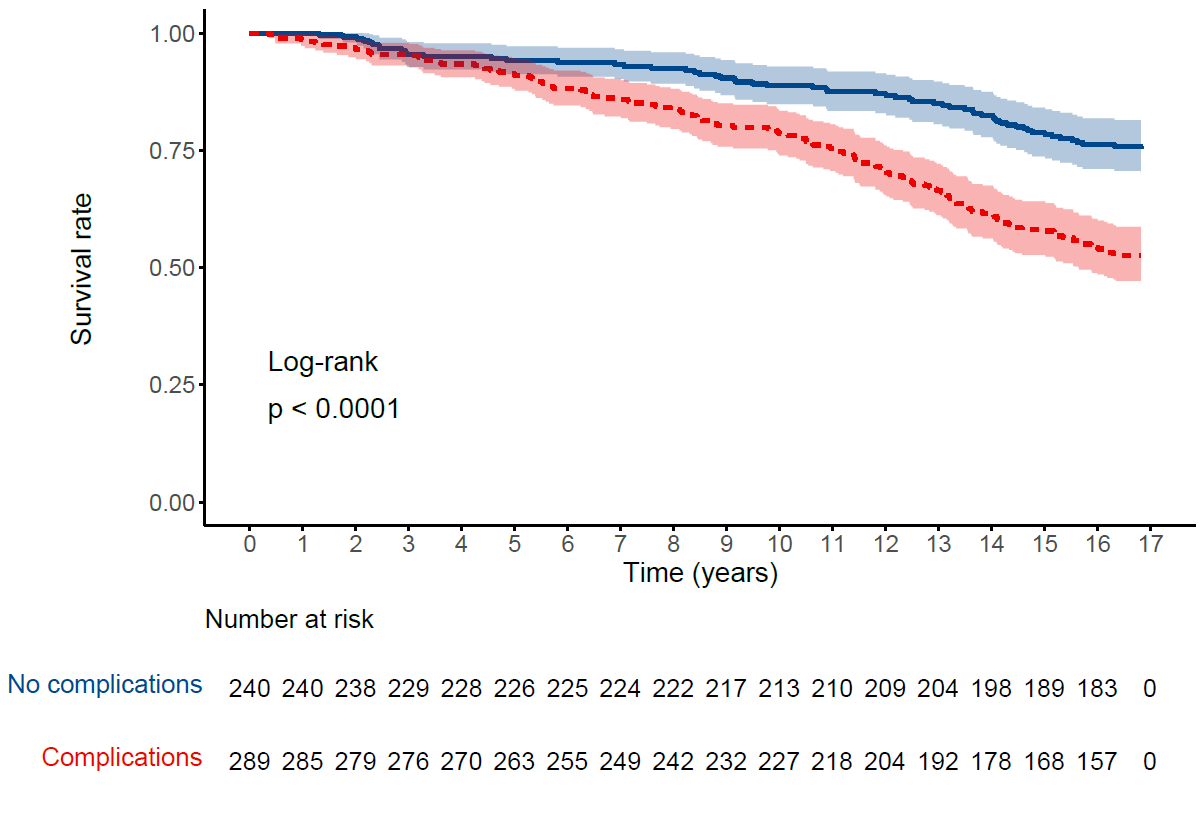


**Table S1.** Two-way ANOVA assessing the effect of sex and the presence of complications on circulating levels of PCSK9.

|  | | **Sum of squares** | | **df** | | **Square mean** | | **F** | | **p** | |
| --- | --- | --- | --- | --- | --- | --- | --- | --- | --- | --- | --- |
| Sex |  | 50210 |  | 1 |  | 50210 |  | 8.728 |  | 0.003 |  |
| T2DM complications |  | 11582 |  | 1 |  | 11582 |  | 2.013 |  | 0.157 |  |
| Sex ✻ T2DM complications |  | 25575 |  | 1 |  | 25575 |  | 4.446 |  | 0.035 |  |
| Residuals |  | 3.020e+6 |  | 525 |  | 5753 |  |  |  |  |  |
|  | | | | | | | | | | | |

PCSK9, proprotein convertase subtilisin/kexin type 9. T2DM, type 2 diabetes mellitus; ✻, interaction effect.

**Table S2.** Circulating levels of PCSK9 in patients with type 2 diabetes mellitus (T2DM) in relation to the different T2DM-related complications.

|  | | **Sum of squares** | | **df** | | **Square mean** | | **F** | | **p** | |
| --- | --- | --- | --- | --- | --- | --- | --- | --- | --- | --- | --- |
| MACE |  | 23315 |  | 1 |  | 23315 |  | 4.1328 |  | **0.043** |  |
| Atherosclerotic vascular disease |  | 2473 |  | 1 |  | 2473 |  | 0.4383 |  | 0.508 |  |
| Nephropathy |  | 30695 |  | 1 |  | 30695 |  | 5.4411 |  | **0.020** |  |
| Neuropathy |  | 3611 |  | 1 |  | 3611 |  | 0.6401 |  | 0.424 |  |
| Retinopathy |  | 9847 |  | 1 |  | 9847 |  | 1.7456 |  | 0.187 |  |
| Residuals |  | 2.933e+6 |  | 520 |  | 5641 |  |  |  |  |  |
|  | | | | | | | | | | | |

MACE, major adverse cardiovascular events. P-values derived from *post-hoc* tests with Tukey's corrections after analysis of covariance (ANCOVA).

Sex, age, and HbA1c (haemoglobin A1C) are considered as covariates.

Significant data are in bold.

**Table S3.** Circulating levels of PCSK9 in patients with type 2 diabetes mellitus in relation to antidiabetic treatments.

|  | | **Sum of squares** | | **df** | | **Square mean** | | **F** | | **p** | |
| --- | --- | --- | --- | --- | --- | --- | --- | --- | --- | --- | --- |
| Metformin |  | 10620.1 |  | 1 |  | 10620.1 |  | 1.8550 |  | 0.174 |  |
| Insulin |  | 774.0 |  | 1 |  | 774.0 |  | 0.1352 |  | 0.713 |  |
| Glinides |  | 3766.9 |  | 1 |  | 3766.9 |  | 0.6580 |  | 0.418 |  |
| Sulphonylureas |  | 2612.0 |  | 1 |  | 2612.0 |  | 0.4562 |  | 0.500 |  |
| Residuals |  | 2.983e+6 |  | 521 |  | 5725.0 |  |  |  |  |  |

PCSK9, proprotein convertase subtilisin/kexin type 9. P-values derived from *post-hoc* tests with Tukey's corrections after analysis of covariance (ANCOVA).

Sex, age, and HbA1c (haemoglobin A1C) are considered as covariates.

**Table S4**. Circulating levels of PCSK9 in patients with type 2 diabetes mellitus in relation to statin therapy.

|  | | Sum of squares | | df | | Square mean | | F | | p | |
| --- | --- | --- | --- | --- | --- | --- | --- | --- | --- | --- | --- |
| Statin therapy |  | 120686 |  | 1 |  | 120686 |  | 21.9994 |  | < .001 |  |
| LDL-C |  | 17669 |  | 1 |  | 17669 |  | 3.2207 |  | 0.073 |  |
| Sex ✻ Statin therapy |  | 3100 |  | 1 |  | 3100 |  | 0.5650 |  | 0.453 |  |
| Residuals |  | 2.842e+6 |  | 518 |  | 5486 |  |  |  |  |  |
|  | | | | | | | | | | | |

LDL-C, low-density lipoprotein cholesterol. ✻, interaction effect.

**Table S5.** Correlation matrix between selected biochemical variables and serum PCSK9 in patients with type 2 diabetes mellitus.

|  |  | **PCSK9** | |
| --- | --- | --- | --- |
| Age | Spearman's rho | 0.069 |  |
|  | p-value | 0.113 |  |
| Fasting insulin | Spearman's rho | 0.109 | * |
|  | p-value | 0.012 |  |
| Fasting glucose | Spearman's rho | 0.127 | ** |
|  | p-value | 0.003 |  |
| HOMA-index | Spearman's rho | 0.159 | *** |
|  | p-value | < .001 |  |
| HbA1c | Spearman's rho | 0.096 | * |
|  | p-value | 0.027 |  |
| Total cholesterol | Spearman's rho | 0.16 | *** |
|  | p-value | < .001 |  |
| LDL-C | Spearman's rho | 0.056 |  |
|  | p-value | 0.203 |  |
| HDL-C | Spearman's rho | 0.007 |  |
|  | p-value | 0.875 |  |
| non-HDL-C | Spearman's rho | 0.168 | *** |
|  | p-value | < .001 |  |
| Remnant cholesterol | Spearman's rho | 0.208 | *** |
|  | p-value | < .001 |  |
| Triglycerides | Spearman's rho | 0.246 | *** |
|  | p-value | < .001 |  |
| hs-CRP | Spearman's rho | 0.06 |  |
|  | p-value | 0.168 |  |
| Apo A1 | Spearman's rho | 0.078 |  |
|  | p-value | 0.075 |  |
| Apo B | Spearman's rho | 0.14 | ** |
|  | p-value | 0.001 |  |
| Waist/hip ratio | Spearman's rho | -0.076 |  |
|  | p-value | 0.083 |  |
| BMI | Spearman's rho | 0.066 |  |
|  | p-value | 0.129 |  |
| ADMA | Spearman's rho | 0.001 |  |
|  | p-value | 0.98 |  |
| SDMA | Spearman's rho | -0.009 |  |
|  | p-value | 0.838 |  |
| PAI-1 | Spearman's rho | 0.171 | *** |
|  | p-value | < .001 |  |
| Blood urea nitrogen | Spearman's rho | 0.045 |  |
|  | p-value | 0.303 |  |
| eGFR | Spearman's rho | -0.077 |  |
|  | p-value | 0.077 |  |
| Creatinine | Spearman's rho | -0.02 |  |
|  | p-value | 0.645 |  |
| Uric acid | Spearman's rho | -0.062 |  |
|  | p-value | 0.156 |  |
| Alkaline phosphatase | Spearman's rho | 0.12 | ** |
|  | p-value | 0.006 |  |
| AST | Spearman's rho | -0.066 |  |
|  | p-value | 0.13 |  |
| ALT | Spearman's rho | -0.039 |  |
|  | p-value | 0.376 |  |
| White blood cells | Spearman's rho | 0.072 |  |
|  | p-value | 0.097 |  |
| Hemoglobin | Spearman's rho | -0.027 |  |
|  | p-value | 0.534 |  |
| HCT | Spearman's rho | 0.036 |  |
|  | p-value | 0.404 |  |
| MCV | Spearman's rho | 0.034 |  |
|  | p-value | 0.433 |  |
| MCH | Spearman's rho | -0.062 |  |
|  | p-value | 0.156 |  |
| MCHC | Spearman's rho | -0.184 | *** |
|  | p-value | < .001 |  |
| RDV-SD | Spearman's rho | 0.089 | * |
|  | p-value | 0.041 |  |
| RDW-CV | Spearman's rho | 0.048 |  |
|  | p-value | 0.273 |  |
| Platelets | Spearman's rho | 0.125 | ** |
|  | p-value | 0.004 |  |
| Platelet-to-large cell ratio | Spearman's rho | -0.039 |  |
|  | p-value | 0.378 |  |
| Ferritine | Spearman's rho | -0.044 |  |
|  | p-value | 0.318 |  |
| Serum iron | Spearman's rho | -0.107 | * |
|  | p-value | 0.014 |  |
| * p < 0.05, ** p < 0.01, *** p < 0.001 | | | |

PCSK9, proprotein convertase subtilisin/kexin type 9
